# Effects of antenatal care and institutional delivery on exclusive breastfeeding practice in northwest Ethiopia: a nested case–control study

**DOI:** 10.1186/s13006-015-0055-4

**Published:** 2015-11-19

**Authors:** Gashaw Andargie Biks, Amare Tariku, Gizachew Assefa Tessema

**Affiliations:** Department of Health Service Management and Health Economics, Institute of Public Health, University of Gondar, Gondar, Ethiopia; Department of Human Nutrition, Institute of Public Health, University of Gondar, Gondar, Ethiopia; Department of Reproductive Health, Institute of Public Health, University of Gondar, Gondar, Ethiopia

**Keywords:** Exclusive breastfeeding, Antenatal care, Institutional delivery, Nested case–control, Ethiopia

## Abstract

**Background:**

For the first six months of life, breast milk is the ideal food to provide adequate quality and quantity of nutrients. Exclusive breastfeeding has a profound effect to reduce the risk of respiratory and gastrointestinal related morbidities as well as all-cause and infection-related neonatal mortalities. Despite the immense benefits of exclusive breastfeeding, the practice is suboptimal in Ethiopia. The aim of this study was to assess whether antenatal care and institutional delivery contributes to mothers' practice of exclusive breastfeeding in rural communities of northwest Ethiopia.

**Methods:**

A community-based nested case–control study was conducted in northwest Ethiopia from November 2009 to August 2011. About 1769 mother-infant pairs were included and followed for six months after birth. Interviews with mothers were conducted in the first week, at 1^st^, 4^th^, and 6^th^ month. Bivariate and multivariate logistic regression were carried out to determine associations between independent variables and exclusive breastfeeding practice.

**Results:**

Of the total respondents, 30.7 % (95 % CI: 27 %, 35 %) of mothers exclusively breastfed their infants. In multivariate analysis, own business activity (AOR= 3.06; 95 % CI: 1.29, 7.25), being a housewife (AOR= 3.41; 95 % CI: 1.28, 9.11), having antenatal care (AOR= 1.32; 95 % CI: 1.01, 1.73), giving birth in a health institution (AOR= 1.29; 95 % CI: 1.02, 1.62), and possessing a microfinance bank account (AOR= 2.35; 95 % CI: 1.80, 3.07) were positively associated with exclusive breastfeeding practice.

**Conclusions:**

Despite underutilization of maternal health services, these services contributed to mothers exclusive breastfeeding practice. Strengthening utilization of antenatal care and institutional delivery would have an added benefit in improving exclusive breastfeeding practice. Moreover involving mothers in business activities is important.

## Background

The World Health Organization (WHO) recommends that infants should be exclusively breastfed for the first six months, and for an additional 18 months or longer, to be breastfed along with complementary foods for the achievement of satisfactory growth and development [1, 2]. In the first six months of age, breast milk is the ideal food to provide adequate quality and quantity of nutrients for infants. Exclusive breastfeeding (EBF) helps infants to strengthen their natural immunological mechanisms. Infants who are not EBF have a higher risk of developing gastrointestinal and respiratory tract infections, otitis media, and atopic eczema than EBF infants [3–6]. Furthermore, EBF is important to promote optimal infant cognitive development [7, 8]. In the long term, EBF can reduce the risk of overweight and obesity and related consequences, such as diabetes mellitus, cardiovascular diseases, and cancers [9]. Early initiation of breastfeeding is associated with nearly 45 % reduction in all-cause and infection-related neonatal mortality [10].

Globally, 60 % of child deaths occur due to inappropriate Infant and Young Child Feeding practices (IYCF) and infectious diseases, from which two-thirds are attributable to suboptimal breastfeeding [1]. Poor IYCF practice is also associated with poor child growth and development, poor school performance, reduced survival, limited work capacity and productivity. These devastating effects are worse in developing countries where basic health services are lacking [2, 11, 12]. Furthermore, in some societies, foods other than breast milk are given to newborn infants, and colostrum is discarded as unclean [13–15]. In countries including Ethiopia, plain water, raw butter, sugar water, fruit juice, honey, sugar-salt water solution, and milk other than breast milk are some of food items given as prelacteal feed [13–19].

Globally, less than 35 % of mothers exclusively breastfeed their infants for the first six months. The problem is very common in sub-Saharan African and other developing countries [1, 10]; 47-57 % of infants less than two months and 25-31 % of infants 2–5 months were exclusively breastfed in Africa, Asia, Latin America, and Caribbean countries [5]. Although breastfeeding seems universal in Ethiopia, it is suboptimal. About 97 % of children have ever been breastfed at some period in their lives, but only 52 % of infants are exclusively breastfed [19]. Studies indicated that maternal related factors, such as maternal education, current marital status, smoking status, place of residence, employment, and economical status of mothers, history of antenatal and postnatal care are associated with EBF practice [20–24].

Antenatal care, a basic component of maternal health care, is an ideal entry point to provide multiple health and nutritional interventions to promote maternal and fetal wellbeing, breastfeeding behaviors [25], and birth preparedness [26]. Additionally, institutional delivery creates better opportunity for neonates to receive skin-to-skin care from their mother, a newborn care practice proven to increase the likelihood of early initiation, exclusive, and prolonged duration of breastfeeding [10, 27]. The WHO recommends at least four objective-based antenatal visits with the first visit in the first trimester (ideally before 12 weeks but not later than 16 weeks), at 24–28 weeks, 32 weeks, and 36 weeks. Each visit should encompass appropriate care to the woman’s overall condition and stage of pregnancy and help her prepare for birth and care for the newborn. As part of the health promotion activity, breastfeeding is one of the key area for counseling pregnant women [28, 29]. However, only 43 % and 10 % of pregnant women in Ethiopia received at least one antenatal care visits and gave birth at health institutions, respectively [19]. There are few available studies on maternal health care and infant feeding in Ethiopia; However, most studies of EBF were cross-sectional surveys and determined retrospectively [30–32]. Therefore this study aimed to assess whether antenatal care and institutional delivery contributes to mothers' exclusive breastfeeding practice in rural communities of northwest Ethiopia.

## Methods

### Study design and setup

A community-based nested case–control study was conducted in Dabat Health and Demographic Surveillance System (DHDSS) site which is located in Dabat district, northwest Ethiopia. The district has an estimated population of 145,458 individuals living in 27 rural and 3 urban *kebeles* (the smallest administration unit). The livelihood of the residents is mainly subsistence farming. The district has two health centers and twenty-nine health posts providing health services for the community. The DHDSS covers ten randomly selected kebeles (three urban and seven rural kebeles) in different ecological zones (high land, middle land, and low land). A total of 46,165 people were living in these kebeles, of which infants comprise about 3 % [33]. Dabat Rural Health Project (the current DHDSS) has been running a Health and Demographic Surveillance System since November 1996. The surveillance site is hosted by the University of Gondar and collects information on vital events like birth, death, migration, and pregnancy registrations and its outcome on a quarterly basis.

### Sample and sample size, and study variables ascertainment

This study was part of a larger prospective follow-up study investigating infant mortality carried out in the DHDSS site. The project included all pregnant women who lived in the DHDSS site (ten kebeles) in the second/third trimester of their pregnancy and were recruited from November 2009 to August 2011. Pregnancy status was confirmed through interview by data collectors. The details of the primary project have been published elsewhere [34, 35].

From the original cohort, mothers who exclusively breastfed their infants for the first six months were selected as cases, while mothers who did not exclusively breastfeed for the first six months were considered controls. A total of 1769 mother-infant pairs (543 cases and 1226 controls) were included in the study. In the original project, mothers were contacted six times in the prospective follow ups: at the first week after birth, 1^st^, 4^th^, 6^th^, 9^th^ and 12^th^ month. For the purpose of the current study, the first four follow-ups: at the first week after birth, 1^st^, 4^th^, and 6^th^ months were used to ascertain exclusive breastfeeding practice. During each follow-up visit, mothers were asked a key question ''did you give any food/fluid for your child starting from date of birth up to today but it does not include any medication or supplements'' and those who responded “No” in all of the four visits qualified as a *“case”*, otherwise considered as a *"control"* in this study. If the mother had given any food/fluid apart from breast milk, the data collectors helped the mother to recall when she had provided this additional food.

The mothers' socio-demographic and economic, household food security status, IYCF knowledge, use of maternal health service, and health care access data were collected at the commencement of the original prospective study. At their first week after birth; birth outcome, place of delivery, maternal and neonatal health care services (vaccination, antenatal and postnatal care) received, neonatal feeding practice (timing of initiation of breastfeeding and any prelacteal foods given), and maternal and neonatal health status related data were collected. At the first month, the following data were collected: health status, any postnatal visit received, number of postnatal visit, exclusive breastfeeding practice, health seeking behavior of the mothers, and any vaccination received. At the four months, exclusive breastfeeding practice, immunization, method of feeding (if any additional food other than breast milk was started), and health status related data were gathered. At six months, exclusive breastfeeding practice, initiation and type of complementary food, dietary diversity, hygiene and sanitation, and other related information were collected. In addition, death was registered at any visit.

### Data collection instrument and quality assurance

Structured, pretested, interviewer-administered questionnaires adapted from the UNICEF multiple indicator cluster survey were employed to collect data [36]. To maintain consistency, the questionnaire was first translated from English to Amharic, the native language of the study area, and back translated to English by professional translators and public health experts. Double data entry was also conducted. The collected data were checked for completeness by the field supervisors and investigators on a daily basis. Seventeen data collectors with high school education and three field supervisors with previous experience in data collection and supervision were recruited. Local informants who were permanent residents of the village were also recruited to assist data collectors and supervisors throughout the study period. They have provided information about completion of pregnancy as soon as possible regardless of gestational age and birth outcome. A five day intensive training about the study objectives, interview techniques, and ethical issues were conducted for data collectors, supervisors, and local informants.

### Operational definitions and study variables

In this study, the exclusive breastfeeding status was ascertained based on the WHO recommendation, starting from the first day of life. An infant was considered to be exclusively breastfed when he or she had received only breast milk with no other liquids (including water) or solids. Early initiation of breastfeeding was defined as infants who initiated breastfeeding within an hour of birth [1].

The outcome variable of this study was exclusive breastfeeding practice with dichotomous category (yes/no). Potential predictor variables were age of the mother, marital status, maternal occupation and educational status, possession of microfinance bank account, place of residence, sex of infant, place of delivery, and antenatal care. Antenatal care for the index child was determined as whether the mother had at least one antenatal visit or not.

### Data analysis

Data were coded and entered into Epi-Info version 3.5.3 and exported to Stata Version 11 software for analysis. Descriptive statistics was used to characterize the study variables. A binary logistic regression was used to identify determinants of EBF practice. Variables with a *p*-value of < 0.2 in bivariate analysis were entered to multivariate analysis to control the possible effect of confounders. The Adjusted Odds Ratio (AOR) with 95 % Confidence Interval (CI) was computed to assess the strength of association, and a p-value of ≤ 0.05 was used to declare the statistical significance in the multivariate analysis.

### Ethical considerations

Ethical clearance was obtained from the Ethical Review Board of University of Gondar (Ref. No RPO 55/338/2001, and Date September 15, 2009) and, submitted to Dabat Research Center/DHDSS site. The objective of the study was explained, and informed verbal consent was obtained from each participant before the interviews took place. Participant records were coded and only accessed by the research team. Participants who were unwilling to participate and wanted to withdraw at any step of the interviews were able to do so without any restriction.

## Results

In total, 1769 mother-infant pairs were included in the study. The mean (± SD) age of the mothers was 27.65 years (± 9.4), with more than two-thirds (68.9 %) in the age range 20–34 years. The majority of mothers were housewives (88.2 %), uneducated (72 %), and from rural kebeles (82.5 %). Nearly one-fifth (18.1 %) of study participants had a microfinance bank account (Table 1). Less than one-third (30.7 %) of mothers had exclusively breastfed their infants for six months. More than one-quarter (25.8 %) of respondents had antenatal care, of which 60.6 % were non-exclusive breastfeeding mothers. Nearly one-third (30.6 %) of mothers gave birth in a health facility, of which 36.4 % were exclusive breastfeeding mothers. Nearly half (47.5 %), one-third (29.8 %), and one-quarter of mothers initiated breastfeeding within one hour, 1–24 h, and after 24 h of delivery, respectively.

**Table 1:** Socio-demographic and economic characteristic of study participants in the rural population of Northwest Ethiopia, 2009–2011

Variables	Exclusive breastfeeding		Total
	Cases	Controls	
	n (%)	n (%)	
Age of mother (years)			
≤19	74 (32.7)	152 (67.3)	226
20–34	372 (30.5)	848 (69.5)	1220
≥35	97 (30.0)	226 (70.0)	323
Marital status of mother			
Married	501 (30.4)	1146 (69.6)	1647
Single, widowed & separated	42 (34.4)	80 (65.6)	122
Educational status of mother			
Uneducated	358 (28.1)	916 (71.9)	1274
Read, write & primary education	86 (35.4)	157 (64.6)	243
Secondary and above	99 (39.3)	153 (60.7)	252
Mothers' occupation			
Farmer	13 (56.5)	10 (43.5)	23
Own business	35 (45.5)	42 (54.5)	77
Housewife	458 (29.4)	1102 (70.6)	1560
Others^a^	37 (33.9)	72 (66.1)	109
Bank account			
Yes	155 (48.4)	165 (51.6)	320
No	388 (26.8)	1061 (73.2)	1449
Place residence			
Urban	212 (39.2)	188 (60.8)	309
Rural	422 (28.9)	1038 (71.1)	1460
Sex of infant			
Male	271 (30.4)	619 (69.6)	890
Female	272 (30.9)	1148 (69.1)	879
Total	543 (30.7)	1226 (69.3)	1769

^a^Student, unemployed

The result of bivariate analysis showed that exclusive breastfeeding practice was associated with maternal educational status, maternal occupation, place of residence, presence of household's microfinance bank account, antenatal care for the index child, and place of delivery (Table 2). However, the multivariate analysis revealed that occupational status of mothers, antenatal care for the index child, place of delivery, and possession of micro-finance bank account were significantly and independently associated with exclusive breastfeeding practice. Accordingly, mothers who were engaged with their own business (self employed) were about three times (AOR 3.06; 95 % CI 1.29, 7.25) more likely to exclusively breastfeed their child; and housewife mothers were also more than threefold (AOR 3.41; 95 % CI 1.28, 9.11) more likely to exclusively breastfeed than their counterparts. Mothers who possessed a microfinance bank account at the household level were more than two times (AOR 2.35; 95 % CI 1.80, 3.07) more likely to exclusively breastfeed than mothers without a bank account. Mothers who had received antenatal care or gave birth at a health institution were more likely to practice exclusive breastfeeding: AOR 1.32 (95 % CI 1.01, 1.73) and AOR 1.29 (95 % CI 1.02, 1.62), respectively (Table 2).

**Table 2:** Factors associated with exclusive breastfeeding practice in the rural population of Northwest Ethiopia, 2009-2011

Variables	Exclusive breastfeeding			
	Cases	Controls	Crude Odds Ratio (95% CI)	Adjusted Odds Ratio (95% CI)
	n (%)	n (%)		
Educational status of mother				
Illiterate	358 (28.1)	916 (71.9)	1	1
Read, write and primary education	86 (35.4)	157 (64.6)	1.66 (1.25, 2.19)	1.21 (0.79, 1.83)
Secondary and above	99 (39.3)	153 (60.7)	1.18 (0.82, 1.70)	1.01 (0.64, 1.57)
Mothers' occupation				
Farmer	13 (56.5)	10 (43.5)	1.56 (0.61, 3.99)	2.57 (0.93, 7.13)
Own business	35 (45.5)	42 (54.5)	3.13 (1.36, 7.19)	3.06 (1.29, 7.25)
Housewife	458 (29.4)	1102 (70.6)	2.53 (1.01, 6.32)	3.41 (1.28, 9.11)
Other^a^	37 (33.9)	72 (66.1)	1	1
Place of residence				
Urban	212 (39.2)	188 (60.8)	1.58 (1.23, 2.04)	1.12 (0.78, 1.61)
Rural	422 (29.9)	1038 (71.1)	1	1
Bank account				
Yes	155 (48.4)	165 (51.6)	2.57 (2.00, 3.29)	2.35 (1.80, 3.07)
No	388 (26.8)	1061 (73.2)	1	1
Antenatal care				
Yes	180 (39.4)	277 (60.6)	1.70 (1.36, 2.12)	1.32 (1.01, 1.73)
No	363 (27.7)	949 (72.3)	1	1
Place of delivery				
Health institution	197 (36.4)	344 (63.6)	1.48 (1.18, 1.81)	1.29 (1.02, 1.62)
Home	346 (28.2)	882 (71.8)	1	1

^a^Student and unemployed

*CI* Confidence Interval

## Discussion

Maternal employment status was an independent predictor of exclusive breastfeeding practice. Mothers who had been engaged in their own business activity and housewives were more likely to exclusively breastfeed their child than mothers who had been engaged in other work activity. The finding was similar to studies reported at Bahir Dar, Ethiopia [30] and three Latin American countries [23]. This might be explained as housewives and women engaged in their own small business are in frequent contact with their child as they spent their time at home or around the home. The study also showed that low maternal knowledge about appropriate IYCF was associated with non-exclusive breastfeeding practice [37].

Mothers who gave birth in a health institution were more likely to practice exclusive breastfeeding than those who gave birth at home. This finding was in agreement with the studies done at Bahir Dar, Ethiopia [30] and Ghana [38]. This could be due to the fact that mothers who give birth in institution have more opportunities to obtain immediate obstetric and postnatal care, such as nutritional education and counseling on the benefit of breastfeeding, correct positioning and attachment, and breast care. It has been noted that postnatal counseling on exclusive breastfeeding is an important determinant factor for increasing EBF practice [31, 32].

Having antenatal care was also a predictor of EBF. Similar findings were reported in Jimma, Ethiopia [39], Rawalpindi [40], and Singapore [24, 41]. Antenatal care attendees may receive nutritional counseling and education as part of the health promotion, such as health benefit of exclusive breastfeeding, and other infant feeding practices during their visits. Health institutions can be good sources of information about breastfeeding and other appropriate feeding practices for mothers [42]. Antenatal care increases not only maternal knowledge about exclusive breastfeeding, but also utilization of institutional delivery [26].

Mothers who possessed a microfinance bank account at the household level were more likely to practicing exclusive breastfeeding than their counterparts. Microfinance bank account is one of the items used in measuring the household wealth index in the Ethiopia Demographic and Health Survey [19], but it is very difficult to use it as an independent household wealth indicator. Therefore the relationship between owning a microfinance bank account and exclusive breastfeeding practice needs further study. However another study in Ethiopia revealed that good economic status increases the likelihood of exclusive breastfeeding [43].

As strength, this study utilized larger sample size, which increases the power of the study, and makes the result more representative. EBF practice was determined prospectively using a 'life-long' or 'since-birth' method using repeated measurements. This nature of measurement provides more accurate estimate of EBF practice than cross-sectional studies, which ascertain EBF using point-in-time data and single 24-hr recall. However this study is not free from limitations, since the date for initiation of foods or drinks other than breast milk was determined retrospectively, we might commit recall bias.

## Conclusion

Employment status, antenatal care, institutional delivery, and having a micro-finance bank account were predictors of exclusive breastfeeding practice. Thus improving utilization of antenatal care and institutional delivery are crucial interventions to increase exclusive breastfeeding practice. Moreover involving mothers in business activities is important.
